# Attitudes and perceptions of nephrology nurses towards dialysis modality selection: a survey study

**DOI:** 10.1186/1471-2369-14-192

**Published:** 2013-09-10

**Authors:** Karthik K Tennankore, Jay Hingwala, Diane Watson, Joanne M Bargman, Christopher T Chan

**Affiliations:** 1 From the Division of Nephrology, Dalhousie University, 5070 Dickson Building 5820 University Avenue, Halifax, NS, Canada; 2 From the Division of Nephrology, University Health Network, Toronto, B3H 2Y9, Canada

**Keywords:** Dialysis, Barriers, Survey, Home dialysis, Peritoneal dialysis, Home hemodialysis

## Abstract

**Background:**

There is a paucity of information about the views of dialysis nurses towards dialysis modality selection, yet nurses often have the most direct contact time with patients. We conducted a survey to better understand nurses’ attitudes and perceptions, and hypothesized that nurses with different areas of expertise would have differences in opinions.

**Methods:**

We administered an electronic survey to all dialysis/predialysis nurses (n = 129) at a large, tertiary care center. The survey included questions about preferred therapy - in-center hemodialysis (CHD), versus home dialysis (home hemodialysis and peritoneal dialysis) and ideal modality mix. Responses were compared between nurses with home dialysis and CHD experience.

**Results:**

The survey response rate was 69%. Both nursing groups ranked patient caregivers and dialysis nurses as having the least impact on patient modality selection. For most patient characteristics (including age > 70 years and presence of multiple chronic illnesses), CHD nurses felt that CHD was somewhat or strongly preferred, while home dialysis nurses preferred a home modality (p < 0.001 for all characteristics studied). Similar differences in responses were noted for patient/system factors such as patient survival, cost to patients and nursing job security. Compared to CHD nurses, a higher proportion of home dialysis nurses felt that CHD was over-utilized (85% versus 58%, p = 0.024).

**Conclusion:**

Dialysis nurses have prevailing views about modality selection that are strongly determined by their area of experience and expertise.

## Background

Home dialysis, which includes peritoneal (PD) and home hemodialysis (HHD), has potential clinical benefits compared to conventional in-center hemodialysis (CHD) including improved survival, quality of life [1-5] and reduced patient and system costs [6,7]. As a result, nephrologists believe home dialysis modalities should constitute a larger proportion of patients than the current trends [8-10]. Despite these perceptions, CHD remains the predominant form of dialysis in North America [11-14]. Numerous factors contribute to a patient’s decision to choose CHD instead of home dialysis [15-21] including the opinions of their social supports [18,19]. Although many studies have examined the perspectives of patients, caregivers, families, and physicians towards modality selection, [8-10,22-24] the opinions of nurses have not been studied in detail. In a multi-center, international survey study that included nurses, participants felt that home dialysis should make up a larger proportion of long-term dialysis therapy, and PD was selected as the ideal, initial dialysis modality for patients [8]. However, this study included attitudes of physicians, administrators, and others professionals in addition to nephrology nurses. Lauder *et al.* exclusively surveyed Australian nephrology nurses about home dialysis, but did not identify if there were differences in opinion between facility-based and home dialysis nurses [25].

The purpose of our study was to survey nurses with different areas of expertise and to identify their attitudes and perceptions towards dialysis modality selection, potential patient barriers, and modality education. We hypothesized that nurses differed in opinion primarily based on their area of expertise.

## Methods

We administered an online survey to all CHD, PD, HHD and pre-dialysis clinic nurses actively working at the University Health Network (UHN), Toronto, Canada. The survey was designed to gauge nursing opinions as to optimal dialysis modality mix, patient and system factors influencing modality selection and patient/nurse modality education. Baseline demographics included age, gender, years of experience, location of nephrology training and Canadian Nursing Association (CNA) certification in nephrology nursing (CNeph(C)). A registered nurse obtains The CNeph(C) after demonstration of competence in nephrology by passing a comprehensive written examination [26-28]. Dialysis modality selection is a core competency of the CNeph(C).

The survey was created within surveymonkey.net online software using a modified Delphi process. The initial domains of the survey were developed after input from a panel consisting of a home dialysis fellow, HHD physician, PD physician and nephrology nurse practitioner with expertise in the area of home dialysis and clinical experience in CHD. These domains included “choices and influences on modality selection”, “patient and system factors that influence modality selection”, “benefits of dialysis location”, “dialysis modality mix” and “dialysis education”. Questions were developed within each domain, and each member of the panel had several opportunities to modify, add or remove individual questions after viewing suggestions from other panel members. This version of the survey was subsequently distributed to a panel of five nurse managers and educators in the areas of HHD, PD, CHD, vascular access and pre-dialysis care. The nurses were instructed to provide open-ended comments and criticisms for each individual survey element, and provide input as to whether the individual questions appropriately covered the domains of interest. The comments were anonymously re-reviewed by the initial panel and the survey was modified to the final version after consensus (web link to final survey: https://www.surveymonkey.com/s/QBVY7TD). The survey was distributed online on 12 Nov 2012, with two follow-up emails at two and five weeks after the survey was opened to improve response rate. The survey was also posted as a web-link on the desktop of computers within the dialysis units to limit non-response due to not viewing emails. A physician without affiliation to either CHD or home dialysis made additional visits to both units to encourage nurses to complete the survey. The UHN research ethics board approved this study.

In the primary analysis, survey responses were compared between CHD nurses and home dialysis nurses (including PD, HHD and predialysis clinic). Results were reported as counts and percentages for categorical variables, and median and interquartile range for non-normally distributed continuous variables. Categorical variables were compared using Fisher’s exact test. Continuous variables were compared with the Wilcoxon rank sum test for comparisons of two groups and the Kruskal-Wallis test for comparisons of three or more groups. A Bonferonni adjustment was used for multiple group comparisons. Statistical analyses were performed using Stata IC, version 12 (StataCorp, College Station, TX). A two-sided P value < 0.05 was considered statistically significant.

## Results

**Figure 1:**
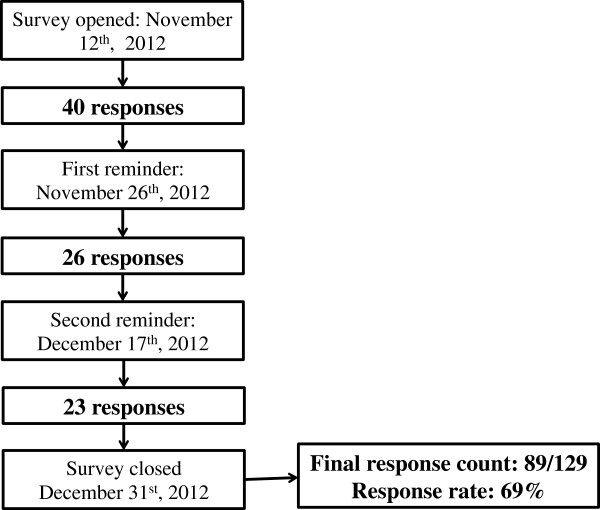
Time sequence of responses.

Of the 129 potential responders, 89 nurses completed the survey (partial or total) resulting in a response rate of 69.0%. The response rate for completed surveys was 60.5% (78/129). The number of responses after each follow-up email is noted in Figure 1. Demographics of the cohort stratified by area of nursing expertise (home dialysis versus CHD) are noted in Table 1. There was a higher proportion of home dialysis nurses with CNeph(C) compared to CHD nurses (84% versus 28%, p < 0.001). The remaining demographics were similar between the two groups.

**Table 1:** Demographic characteristics of the survey responders*

**Variable**	**Home dialysis nurses (n = 25)**	**CHD nurses (n = 64)**	**P**
Age range in years, n (%)			0.14
31-40	3 (12)	4 (6)	
41-50	12 (48)	27 (42)	
51-60	6 (24)	29 (45)	
>60	3 (12)	4 (6)	
No response	1 (4)	0 (0)	
Female gender, n (%)	24 (96)	60 (94)	1.00
Years of nephrology nursing, n (%)			0.13
1-5	1 (4)	3 (5)	
6-10	3 (12)	20 (31)	
11-15	2 (8)	14 (22)	
16-20	6 (24)	8 (13)	
>20	13 (52)	19 (30)	
Location of initial nephrology			0.58
training, n (%)			
Canada	21 (84)	54 (84)	
United States	0	0	
Europe	0	3 (5)	
Other	4 (16)	7 (11)	
CNeph(C) certification, n (%)	21 (84)	18 (28)	<0.001

*Percentages may not add to 100 due to rounding.

### Influence on modality selection

Nurses were asked to identify which group or individual had the most influence on a patients’ choice of dialysis modality. Physicians were ranked as having the most impact by home dialysis nurses (in 87% of responses) and CHD nurses (in 57% of responses). In contrast, dialysis nurses were ranked as having the least impact in 48% and 38% of responses for home dialysis and CHD nurses, respectively.

### Personal choice of modality

When nurses were asked what modality they would select for themselves if they required dialysis, 80% of home dialysis nurses preferred a home modality (either PD or HHD) compared to 52% of CHD nurses. There were differences amongst home dialysis nurses, such that HHD nurses preferred HHD (in 86% of responses), while PD nurses preferred PD (in 79% of responses).

### Modality preference

Nurses were asked if home dialysis or CHD was preferred when given a list of several patient and system factors. Home dialysis nurses had statistically significant differences in opinion compared to CHD nurses for all factors studied (Table 2). A sensitivity analysis was conducted on the 36 nurses who completed their CNeph(C). Statistically significant differences in opinion persisted for all factors except for patient quality of life (median Likert rating of “home dialysis is strongly preferred” for both home dialysis nurses and CHD nurses, p = 0.12).

**Table 2:** Likert rating response for patient characteristics and system/patient factors stratified by home versus CHD nursing groups (median rating, interquartile range)*

	**Home dialysis nurses**	**CHD nurses**	**P**
Patient characteristics			
Poor socioeconomic status	2, 1-4	5, 4-5	<0.001
Multiple chronic illnesses	2, 1-3	5, 4-5	<0.001
No education after high school	2, 1-3	4, 4-5	<0.001
Age greater than 70 years	2, 1-3	4, 3-5	<0.001
English not primary language	2, 2-2	4, 3-5	<0.001
Working or studying part-time or full-time	1, 1-1	2, 1-3	<0.001
No patient caregivers or social supports	2, 1-3	5, 4-5	<0.001
Patient factors			
Better patient quality of life	1, 1-1	1, 1-4	0.01
Better patient survival	1, 1-1	3, 1-5	0.001
Lower cost to patients	1, 1-2	4, 2-5	0.003
Lower risk of catastrophic events to patients	2, 1-3	5, 3-5	<0.001
System factors			
Lower cost to the healthcare system	1, 1-1	2, 1-5	<0.001
Employment and job security for current dialysis nurses	2, 1-3	5, 4-5	<0.001

*Likert rating: 1: Home dialysis strongly preferred, 2: Home dialysis somewhat preferred, 3: Neither CHD or home dialysis preferred, 4: CHD somewhat preferred, 5: CHD strongly preferred.

### Ideal modality distribution

Nurses were asked what they felt would be the ideal proportion of patients that should receive each modality at the UHN. While the perceived ideal proportion of self-care hemodialysis and HHD were similar comparing each nursing group, significant differences in the perceived ideal proportion of CHD and PD were noted (Table 3). Overall, 85% of home dialysis nurses versus 58% of CHD nurses felt that the ideal proportion of patients receiving CHD should be lower than current perceived proportions (p = 0.024). Exclusion of outliers (those selecting a single modality 100% of the time) or restriction to only those nurses with CNeph(C) certification did not significantly alter the results (data not shown).

**Table 3:** Perceived current and ideal proportion of each dialysis modality stratified by nursing group (median proportion, interquartile range)

	**CHD nurses**	**HHD nurses**	**PD nurses**	**Predialysis CKD nurses**	**P**
CHD proportion					
Current	55 (50–60)	50 (50–70)	52.5 (30–60)	60 (57.5-60.5)	0.49
Ideal	45 (30–60)	40 (25–50)	12.5 (0–25)	37.5 (27.5-50)	Comparing CHD to PD nurses: <0.001*
HHD proportion					
Current	10 (10–20)	14 (10–20)	15 (10–15)	16.5 (12–19.5)	0.83
Ideal	20 (10–30)	25 (25–30)	25 (20–30)	20 (20–22.5)	0.50
Self-Care proportion					
Current	10 (5–10)	5 (1–10)	5 (0–5)	7.5 (4–12.5)	0.04
Ideal	10 (5–20)	10 (10–10)	10 (5–10)	10 (10–10)	0.77
PD proportion					
Current	20 (10–25)	23 (10–25)	27.5 (24–30)	17.5 (15–25)	0.05
Ideal	20 (10–25)	20 (10–25)	50 (35–55)	30 (20–40)	Comparing CHD to PD nurses: <0.001* Comparing HHD to PD nurses: 0.001*

*Statistically significant (Bonferonni adjusted P = 0.008).

### Perception of modality education for patients and nurses

Both home dialysis and CHD nurses felt that patients would benefit from further education about dialysis modalities. Both nursing groups were well informed about all dialysis modalities, but also felt they would benefit from additional education, primarily in the form of a practical continuing education/in-service (Table 4).

**Table 4:** Likert rating responses for questions surrounding patient and nurse education stratified by CHD versus home dialysis nursing group (median rating, interquartile range)*

**Questions**	**Home dialysis nurses**	**CHD nurses**	**P**
Patients are well informed of all modalities once they start dialysis	2.5 (2–4)	3 (2–4)	0.58
Patients would benefit from further modality education after they start dialysis	4 (3–5)	4 (4–5)	0.82
I am well informed of all dialysis modalities	4.5 (4–5)	4 (3–4)	0.009
I educate patients about dialysis modalities	4 (3–5)	4 (3–4)	0.41
I would benefit from further education about dialysis modalities	4 (4–4)	4 (4–5)	0.05

*Likert rating: 1: Strongly disagree, 2: Disagree, 3: Neither agree or disagree 4: Agree, 5: Strongly agree.

## Discussion

To our knowledge, this survey study is the first to compare the opinions of home dialysis and CHD nurses towards dialysis modality selection. We identified that both CHD and home dialysis nurses perceived that they had a limited impact on patient decisions about dialysis modality selection. When nurses were given a list of patient characteristics, patient factors, and system factors, there were consistent differences in opinion as to preferred modality. The perceived ideal dialysis modality mix was different comparing PD to CHD and HHD nurses. Finally, there was a desire from both home dialysis nurses and CHD nurses for further modality education.

Dialysis nurses perceived that they had little influence on patient modality selection, but they may be underestimating the weight of their influence on patient decisions. It has been shown that the transference of healthcare workers’ opinions can unknowingly influence patient decisions [29]. Their perception of their own minimal impact may be explained by the timing at which they meet patients. Under usual conditions, dialysis nurses are exposed to patients after they have received modality education from physicians and predialysis chronic kidney disease (CKD) clinic nurses. Therefore, they may feel that sufficient education has already been provided to patients. The perception of minimal impact may also relate to the absence of a formal training program to teach dialysis nurses how to educate patients. Although nephrology nurses can be effective at guiding modality selection [30-32], traditionally, the role of dialysis nurses has not primarily been patient education.

There was a marked difference in the perception of preferred modality comparing CHD to home dialysis nurses, even among those who were taught about dialysis modalities through the CNeph(C). We believe this is strongly related to practical experience. While the UHN practices a “home dialysis first” philosophy, CHD nurses are frequently exposed to PD and HHD patients with technique failure, while home dialysis is sometimes used as “rescue” therapy for patients who are not managing on CHD. This differential exposure may skew perceptions about ideal modalities for patients and the ideal modality distribution for the UHN. In keeping with this hypothesis, a qualitative study demonstrated that practical experience was the most influential factor in nursing practice patterns [33]. As nurses indicated that they preferred practical continuing education (CE) to learn about dialysis modality selection, providing them with hands-on exposure to both the CHD and home dialysis unit (i.e. rotations in other modality units or clinics) may lead to more unified attitudes about dialysis modality selection.

Despite the promotion of home dialysis modalities at our center, only PD nurses felt that more PD usage would be ideal. This is contrasted by survey studies of nephrologists from Canada, United States, and the United Kingdom, where PD was consistently thought to be underutilized [8,9,34]. The difference in opinion may exist for many reasons, with practical experiences once again playing a major role. Another potential influence is perceived job security, a systemic factor that was identified in this study. A fear of changes or restrictions in resource allocation (including job loss) may influence nurses’ perceptions of ideal modality mix.

Our study had a number of important strengths. With respect to the study design, we had input from several experts in the area of dialysis, which improved the face validity and content validity of our survey. Questions were objective in nature, limiting the potential for expert panel bias towards home dialysis or CHD. Finally, we achieved a response rate of 69%, which is higher than other opinion surveys in the area of dialysis modality selection, and mitigates the potential for nonresponse bias. The majority of non-respondents came from the CHD nursing group, however, so nonresponse bias is still a possibility. There are other limitations to our study. As this survey was conducted in a single tertiary care center, the results may not be generalizable to other dialysis centers with different nursing characteristics. A larger multicenter study could provide further insight in this regard. With respect to the survey design, while most of the individual survey items were objective, nurses may have interpreted the subjective items, (such as socioeconomic status) variably. While our response rate was good, we still had a relatively small sample size. Therefore, robust inferences about nursing opinion and modality selection cannot be made. This study did not specifically identify whether patients’ choice of modality was influenced by the preference(s) of nurses. Identifying the impact of nursing opinions on patient perceptions of dialysis modalities would be valuable in future studies. Finally, our results may not reflect current practices by the respondents, and may not encompass all factors influencing modality decisions.

## Conclusions

We identified a marked difference in opinion between CHD and home dialysis nurses surrounding dialysis modality selection. Future studies should examine whether these opinions can be modified, and how they influence patient modality selection.

## Abbreviations

(CHD): In-center hemodialysis; (PD): Peritoneal dialysis; (HHD): Home hemodialysis; (UHN): University health network; (CNA): Canadian nursing association; (CNeph(C)): Certification in nephrology nursing; (CKD): Chronic kidney disease.

## Competing interests

The authors declare that they have no competing interests.

## Authors’ contributions

All five authors contributed to the conception, design analysis and data interpretation. KT and JH drafted the original article, and CC, JB and DW provided article revisions. Final approval of the version to be published was given by all five authors. All authors read and approved the final manuscript.

## Pre-publication history

The pre-publication history for this paper can be accessed here:

http://www.biomedcentral.com/1471-2369/14/192/prepub
